# Projecting potential distribution of *Eucryptorrhynchus scrobiculatus* Motschulsky and *E. brandti* (Harold) under historical climate and RCP 8.5 scenario

**DOI:** 10.1038/s41598-017-09659-3

**Published:** 2017-08-22

**Authors:** Yingchao Ji, Wen Luo, Ganyu Zhang, Junbao Wen

**Affiliations:** Beijing Key Laboratory for Forest Pests Control, College of Forestry, Beijing Forestry University, Beijing, 100083 China

## Abstract

*Ailanthus altissima* (Mill.) Swingle and its variant *A. altissima* var. Qiantouchun are notorious invasive weeds. Two weevils, *Eucryptorrhynchus scrobiculatus* (ESC) and *E. brandti* (EBR) are considered as candidates for biological control of *A. altissima*. The aim of this study was to model the potential distributions of ESC and EBR using CLIMEX 4.0. The projected potential distributions of ESC and EBR included almost all current distribution areas of *A. altissima*, except Southeast Asia. Under historical climate, potential distribution area of EBR is larger than that of ESC, 46.67 × 10^6^ km^2^ and 35.65 × 10^6^ km^2^, respectively. For both ESC and EBR, climate change expanded the northern boundary of potential distributions northward approximately 600 km by the middle of 21st century, and 1000 km by the end of 21st century under RCP 8.5. However, the suitable range decreased to the south in the Southern Hemisphere because of heat stress. The modelled potential distributions of ESC and EBR in the United States demonstrated that the climate was suitable for both weevils. Therefore, considering only climate suitability, both ESC and EBR can be considered as potential biological control agents against *A. altissima* with some confidence that climatic conditions are likely suitable.

## Introduction

Tree-of-heaven *Ailanthus altissima* (Mill.) Swingle and its variant *A. altissima* var. Qiantouchun are planted throughout China as an ornamental plant that also plays an important role in environmental applications^1^. However, it is classified as a noxious weed and invasive species in many countries for its rapid growth, allelopathic effects, extensive root system and ability to reproduce quickly via diaspores and clonal growth^2–4^. *A. altissima* not only out-competes native vegetation but also causes damage to roadways, sidewalks, sewer structures, and orchards with its extensive root system^5^, which is now distributed over all continents from tropical and subtropical areas to temperate and arid regions worldwide^6, 7^. In the United States, *A. altissima* is spreading rapidly, and now widely distributed across 42 states in the United States from Washington to New England and south to Florida, Texas, and Southern California^5, 8^. Furthermore, *A. altissima* is widely naturalized in Europe^9^ and has been introduced into South America, Africa, and Oceania as an invasive alien plant^3, 10^.

In contrast to the invaded areas, many trees of *Ailanthus altissima* die from the attack of two weevils, *Eucryptorrhynchus scrobiculatus* Motschulsky, 1854 (ESC) and *E. brandti* (Harold), 1880 (EBR), which are the most destructive pests of tree-of-heaven and only do damage to tree-of-heaven in China^11, 12^. ESC and EBR are widely distributed in northern, central, northwestern, and southeastern China, and were listed as “National Forest quarantine pests” in 2003, 2013 by National Forestry Bureau in China^13^. The larvae of ESC feed on the roots of *A. altissima* and cause the flow of resin from the injured site on the root, generating a complex of glue and soil around the root. The larvae of EBR drill into stems of *Ailanthus altissima* and leave many holes after adult emergence, and with the major disruption of nutrient and water transport, weakened *A. altissima* can die. Additionally, adults of ESC and EBR feed on the buds, shoots, and leaves of *A. altissima* to satisfy nutritional supplement requirements for reproduction. In areas of Huaibei in China, the proportions of damaged and dead *A. altissima* caused by EBR can exceed 80% and 37%, respectively, of trees planted on both sides of the road^14, 15^. Compared with EBR, ESC causes relatively less damage and death to tree-of-heaven at 15% and 7%, respectively, in second-generation farmland shelterbelt networks in Ningxia Hui Autonomous Region, China^16^.

Because of the serious damage caused by ESC and EBR to *A. altissima* in China, both ESC and EBR are considered as candidates for biological control of tree-of-heaven. In 2004, EBR was identified in China and introduced into the United States as a potential biological control agent, followed by much research to evaluate the quarantine status for EBR^17–19^. Additionally, as a closely related species of EBR, ESC also damages *A. altissima* by feeding on the roots. However, whether these weevils can survive in the areas in which the tree-of-heaven has invaded and curb excessive growth remains to be determined.

In recent years, global climate warming has become a common concern because of the considerable effects on the survival, development, and distribution of animals and plants^20–22^. As a result of global climate warming, the geographical distributions and climatically suitable areas for insects will likely expand into areas that are not currently suitable for survival and development based on current climate data^23, 24^. The greenhouse gas emission scenarios used in Fifth Assessment Report (AR5) for estimating climate change are Representative Concentration Pathways (RCPs), including RCP 2.6 with a CO_2_ concentration reaching 421c, RCP 4.5 (538 ppm), RCP 6.0 (670 ppm), and RCP 8.5 (936 ppm)^25^. Based on the Coupled Model Intercomparison Project Phase 5 (CMIP5) models, global warming is projected to continue. Relative to 1986–2005, the global mean surface temperature by the end of the 21st century will increase by 0.3–4.8 °C^26^. Currently, global climate modelling data is used in climate envelope models to estimate climatically suitable areas for animals and plants. The effect of climate change on pest risk is an aspect of pest risk analysis that has been recognised for some time^27, 28^, but has yet to gain much traction in formal risk assessments^29^. Furthermore, the potential areas of suitability for ESC and EBR can also possibly expand to other regions under a warming global climate. Therefore, the study of potentially suitable for ESC and EBR under global warming scenarios is essential.

Species distribution models have been developed that are used to project the potential geographical distributions of species, which include the bioclimatic niche model (Bioclim^30^, CLIMEX^31^), and ecological niche model (Domain^32^, GARP^33^, MaxEnt^34^). These models such as Bioclim, Domain, GARP and MaxEnt attempt to characterise the environment occupied by the species. However, CLIMEX simulates the mechanisms that limit species’ geographical distributions and determine their seasonal phenology, and to some extent their relative abundance. CLIMEX describes how the species responds to climatic variables at appropriate temporal scales, rather than focus on describing the relationship between the occurrences of the species with respect to static environmental covariates. It was first described by Sutherst and Maywald in 1985^35^, and then several enhancements and further caveats and insights into using the model are described in a series of publications^36–38^. The CLIMEX model is used extensively in research on potential distributions of species, including those of *Lantana camara* L.^39^, *Melaleuca quinquenervia* ^40^, *Cydia pomonella* (L.)^41^, *Rhynchophorus ferrugineus* (Olivier)^42^, and *Eichhornia crassipes* ^43^.

In this study, we applied CLIMEX 4.0 (Hearne Scientific Software, Melbourne, Australia, http://www.hearne.software/Software/) and ArcGIS 10.1 (ESRI, Redlands, CA, USA, http://resources.arcgis.com/en/home/) to assess the sensitivity of the potential distributions of ESC and EBR to future climate scenario RCP 8.5 in the middle and latter 21st century.

## Results

### Potential distribution areas under historical climate conditions

**Figure 1:**
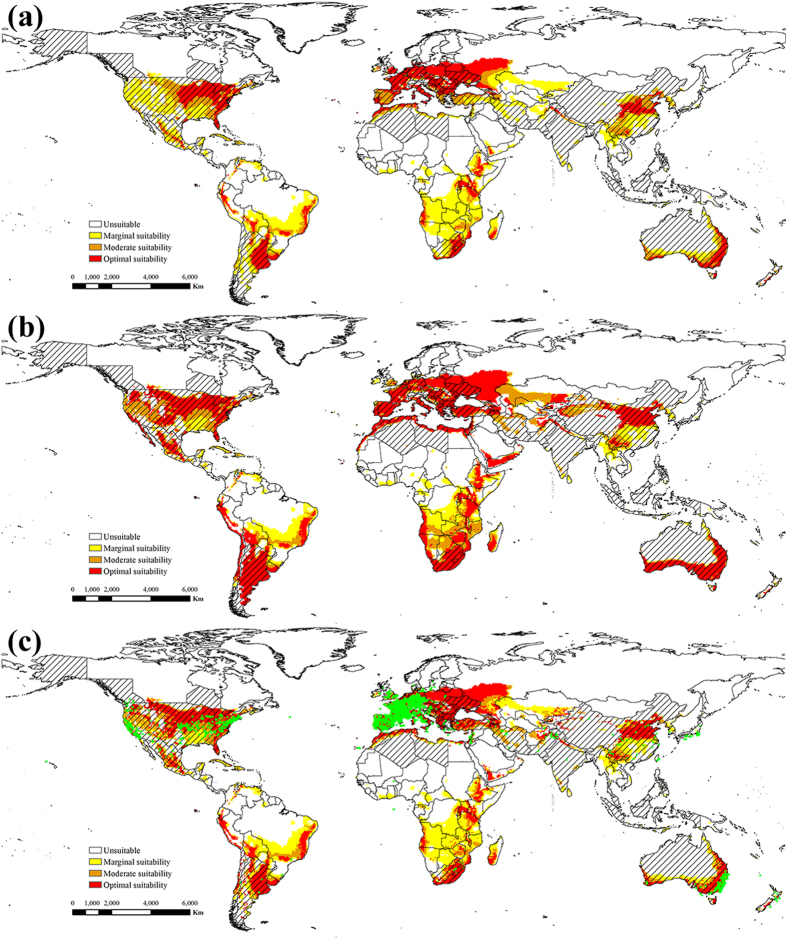
The projected global climate suitability for ESC under the historical climate, (**a**) without irrigation, (**b**) with 2.5 mm in the summer and 1.5 mm in the winter top-up irrigation and (**c**) with a composite risk irrigation scenario (where areas are not under irrigation, the EI of the natural rainfall scenario is mapped, while with areas under irrigation the EI of the irrigation scenario is mapped), using the CLIMEX EI. Slash indicate the distribution countries of tree-of-heaven. Green points indicate the distribution locations of tree-of-heaven. Yellow indicates areas of marginal suitability (0 < EI ≤ 10); Orange indicates areas of moderate suitability (10 < EI ≤ 20); Red indicates areas of optimal suitability (EI ≥ 20). The map in this figure was generated by ArcGIS 10.1 (ESRI, Redlands, CA, USA, http://resources.arcgis.com/en/home/).

**Figure 2:**
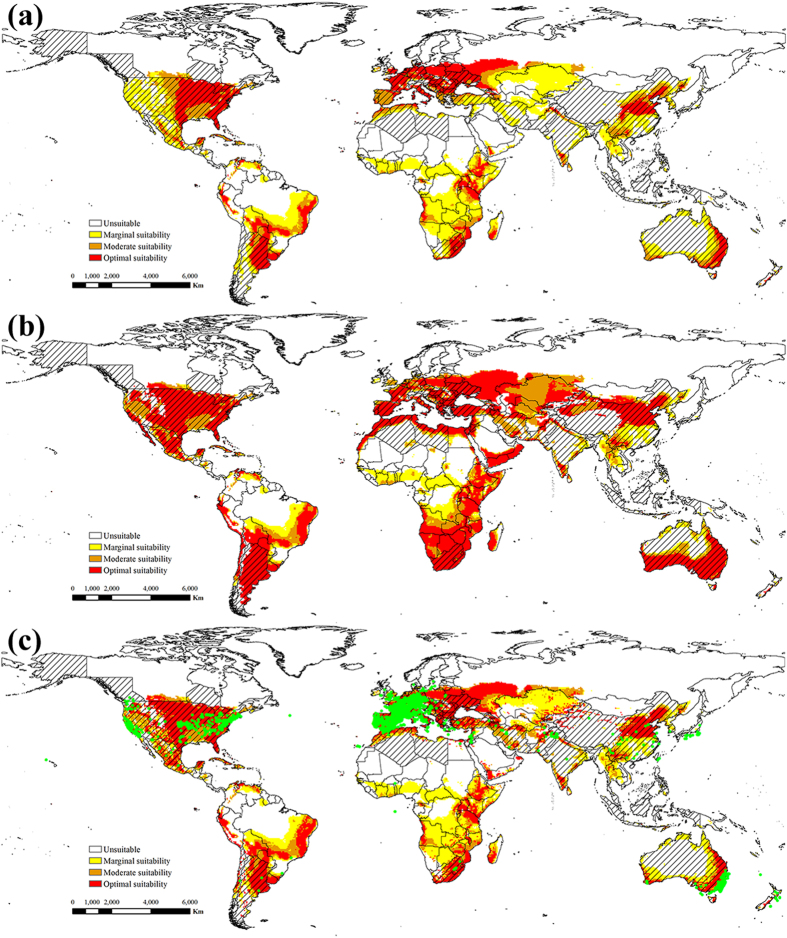
The projected global climate suitability for EBR under the historical climate, (**a**) without irrigation, (**b**) with 2.5 mm in the summer and 1.5 mm in the winter top-up irrigation and (**c**) with a composite risk irrigation scenario (where areas are not under irrigation, the EI of the natural rainfall scenario is mapped, while with areas under irrigation the EI of the irrigation scenario is mapped), using the CLIMEX EI. Slash indicate the distribution countries of tree-of-heaven. Green points indicate the distribution locations of tree-of-heaven. Yellow indicates areas of marginal suitability (0 < EI ≤ 10); Orange indicates areas of moderate suitability (10 < EI ≤ 20); Red indicates areas of optimal suitability (EI ≥ 20). The map in this figure was generated by ArcGIS 10.1 (ESRI, Redlands, CA, USA, http://resources.arcgis.com/en/home/).

Under historical climate conditions, potential distributions worldwide of ESC and EBR were projected from the known distributions and the relevant biological data (Figs 1 and 2). Modeled suitability of the climate for ESC and EBR fit the known distributions in China very well. All the distribution points of ESC and EBR in China accord with the modelled suitable range. A total of 26.70% of the world’s land mass (excluding Antarctica), or 35.65 × 10^6^ km^2^, is climatically suitable for ESC, and for EBR, 34.95% or 46.67 × 10^6^ km^2^ of the global landmass was suitable (Table 1).

**Table 1:** The climatically suitable area (EI > 0) on each continent for ESC and EBR under the historical climate, expressed as an area (10^6^ km^2^) and as a percentage of the total land area per country or region, and the percentage change in the middle and latter of the 21st century under future climate change scenario RCP 8.5.

Areas	Area with EI > 0 under historical climate				Percentage of total land areas with EI > 0 under RCP 8.5			
	ESC		EBR		ESC		EBR	
	Total area (10^6^ km^2^)	% total land areas	Total area (10^6^ km^2^)	% total land areas	2046–2065	2081–2100	2046–2065	2081–2100
China	3.31	34.47	4.18	43.51	37.52	33.04	48.44	53.31
USA	5.48	58.57	6.19	66.16	52.79	43.83	68.53	64.81
Asia	6.46	14.83	10.96	25.14	14.53	14.97	26.68	28.52
Africa	10.64	36.00	14.09	47.67	23.88	12.83	41.30	32.47
Australia	2.53	33.18	3.67	48.22	23.59	14.97	37.85	27.40
Europe	4.75	47.27	4.87	48.50	61.58	71.50	62.27	72.37
NorthAmerica	5.40	22.17	6.51	26.73	20.88	19.69	30.29	33.59
SouthAmerica	7.58	41.87	9.04	49.94	31.09	23.13	50.57	40.51
NewZealand	0.07	24.12	0.07	24.71	33.53	34.12	33.53	35.29
World	35.65	26.70	46.67	34.95	24.13	21.91	36.21	35.66

Under historical climate conditions, the following areas were projected to possess a suitability climate for ESC (Fig. 1): North Ameica (United States, Mexico, Cuba, Dominicana), South America (Peru, Chile and Bolivia, southern Brazil, Argentina, and Uruguay), Europe (excluding Norway, Sweden, Finland, Iceland, and most of Russia), Africa (Morocco, Tunisia, northern Algeria and Libya, Ethiopia, Kenya, Tanzania, Zimbabwe, Mozambique, Angola, Botswana, South Africa, Madagascar), the eastern and southern coasts of Australia, Aisa (southwestern and eastern of China, southern India, Korea, Kazakhstan, Iran, Afghanistan). For EBR, the suitability range is similar to ESC, but the distribution area is larger than ESC (Fig. 2 and Table 1).

The potential global distribution of ESC and EBR in the absence of irrigation are shown in Figs 1a and 2a, respectively. The potential global distribution when irrigation scenarios of 2.5 mm day^−1^ in the summer and 1.5 mm day^−1^ in the winter are added as a top-up to natural rainfall are shown in Fig. 1b and Fig. 2b. Figures 1c and 2c gives a composite potential distribution map, based on areas across the globe considered to be under irrigation according to Siebert *et al*.^44^. There are significant differences in the potential distribution areas under irrigation scenario and non-irrigation scenario. The difference is mainly concentrated in the areas of northwestern China, western United States, southern Australia, southern South America, southern Africa and Western Asia.

Additionally, of note, the projected potential distributions of ESC and EBR are almost covered by the distribution of tree-of-heaven, except for Southeast Asia (Figs 1c and 2c). The concordance of the projected distribution of potential biological control agents of ESC and EBR with the current distribution of tree-of-heaven was analyzed. 98.75 percent or 14996 of distribution points of tree-of-heaven were located in the projected potential distributions of ESC, and 98.97 percent for that of EBR. Therefore, considering only climate suitability, both ESC and EBR can be introduced into the United States as potential biological control agents against *A. altissima* with some confidence that climatic conditions are likely suitable.

### Potential distribution areas under climate change scenario RCP 8.5

**Figure 3:**
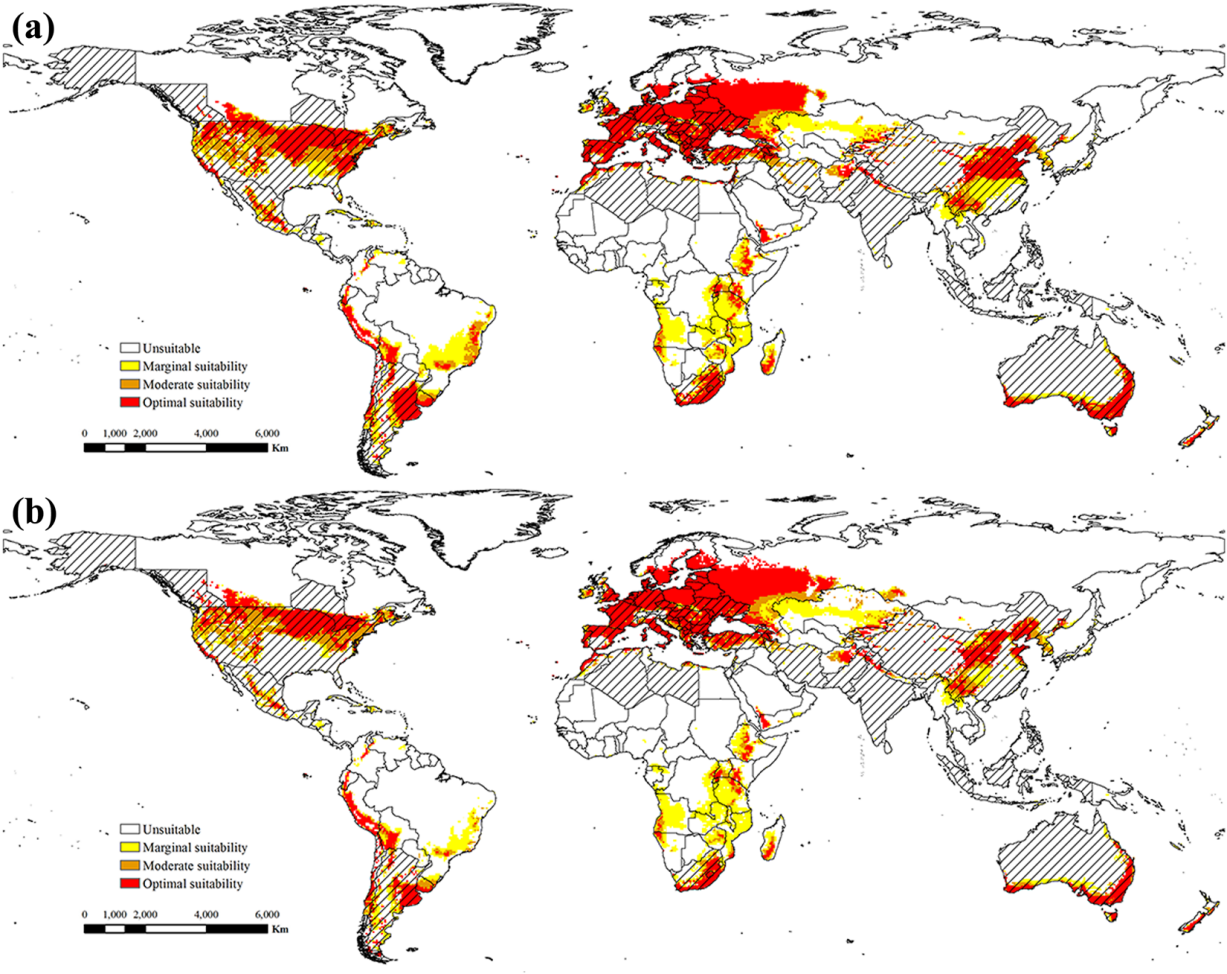
The projected global climate suitability for ESC under climate change scenario RCP 8.5, (**a**) the middle of 21st century (2046–2065), (**b**) the end of 21st century (2081–2100). Slash indicate the distribution countries of tree-of-heaven. Yellow indicates areas of marginal suitability (0 < EI ≤ 10); Orange indicates areas of moderate suitability (10 < EI ≤ 20); Red indicates areas of optimal suitability (EI ≥ 20). The map in this figure was generated by ArcGIS 10.1 (ESRI, Redlands, CA, USA, http://resources.arcgis.com/en/home/).

**Figure 4:**
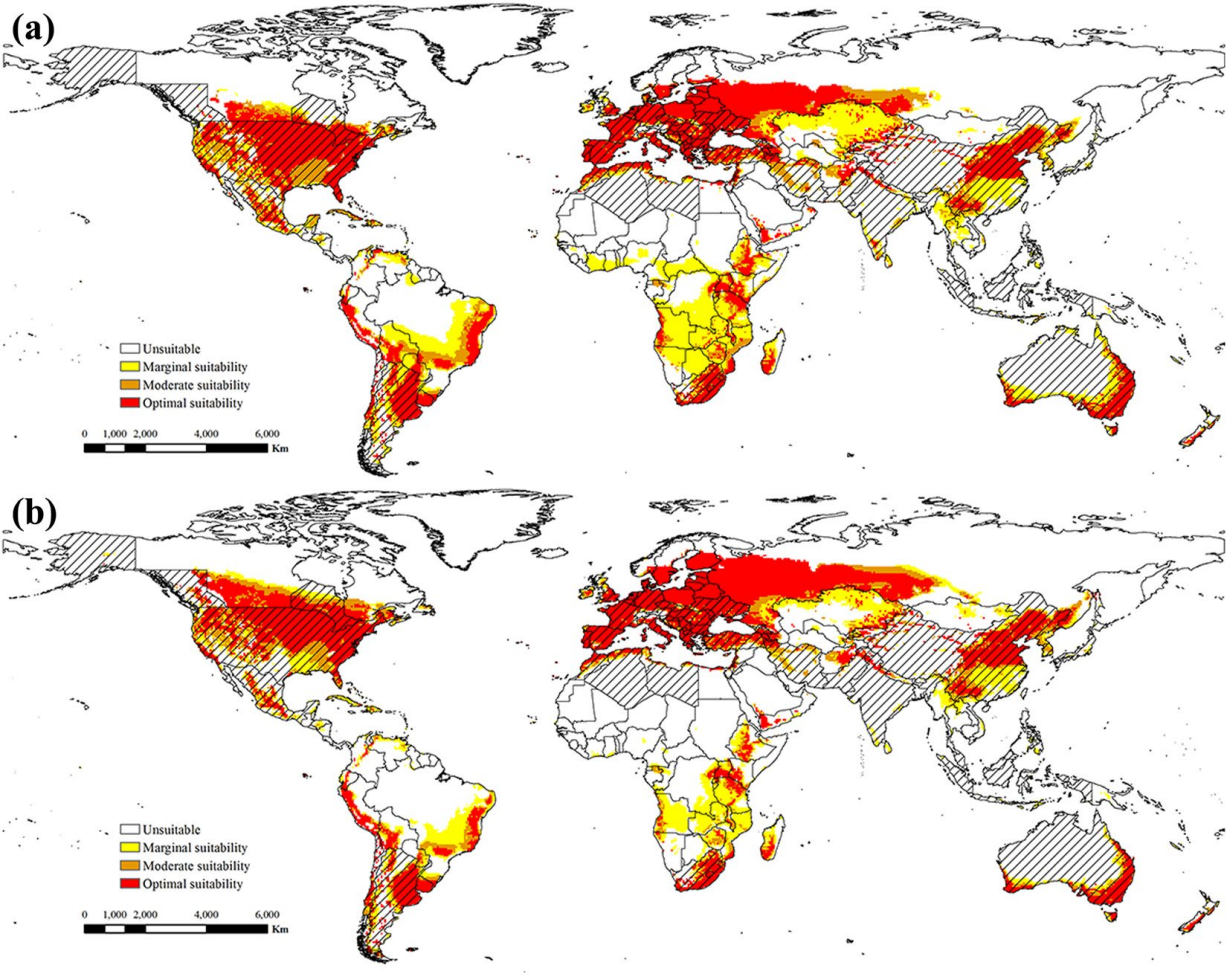
The projected global climate suitability for EBR under climate change scenario RCP 8.5, (**a**) the middle of 21st century (2046–2065), (**b**) the end of 21st century (2081–2100). Slash indicate the distribution countries of tree-of-heaven. Yellow indicates areas of marginal suitability (0 < EI ≤ 10); Orange indicates areas of moderate suitability (10 < EI ≤ 20); Red indicates areas of optimal suitability (EI ≥ 20). The map in this figure was generated by ArcGIS 10.1 (ESRI, Redlands, CA, USA, http://resources.arcgis.com/en/home/).

With global warming, the potential global distributions of ESC and EBR were projected from the AR5 climate change scenario 8.5 in the middle and latter 21st century (Figs 3 and 4). As a result, for both ESC and EBR, climate change expanded the northern boundary of potential distributions northward approximately 600 km by the middle of 21st century, and 1000 km by the end of 21st century under climate change scenario RCP 8.5.

For ESC (Fig. 3), the potential areas of distribution were projected to increase significantly in Europe, little change in Asia, and reduce slightly in North America. However, potential suitable areas in South America, Africa, and Australia were reduced significantly. With the increase in greenhouse gas emissions, the expansions occurred because climate change in these regions was sufficient to overcome cold stress limitations, whereas the reductions might be a consequence of lethal heat stress because of increasing temperatures. Suitable areas for ESC in North America increased in Canada, although suitable areas became unsuitable in the southern United States and on the coast of Mexico. In Europe, the areas of potential distribution increased significantly, with the range from southern Spain to central Finland. Areas with significant reductions in potential distribution were concentrated in Morocco, Tunisia, Algeria, Libya, Botswana, South Africa, Zambia and Mozambique in Africa. In Asia, the climate for ESC was not suitable in India by the end of 21st century. In Australia, areas of potential distribution decreased to the southwest.

Areas of potential distribution were projected to increase slightly for EBR globally (Fig. 4), but significant increases were projected in Europe and North America. Additionally, areas of potential distribution decreased significantly in Africa and Australia in the middle and latter 21st century. In North America, based on the potential distribution areas under the historical climate, these areas were projected to extend in range to northern Canada by overcoming cold stress limitations. In South America, areas of potential distribution were almost unchanged, and only the degree of suitability changed. In Europe, areas of potential distribution were projected to increase significantly, extending the range from southern Spain to central Finland. In Africa, areas of potential distribution were concentrated in southern Africa from approximately 10°N to 33°S. In Asia, under climate change scenario RCP 8.5, the potential range was projected to expand to the north, reaching approximately 70°N. Similar to those of ESC, areas of potential distribution decreased to the southwest in Australia.

### Potential distribution areas of ESC and EBR in the United States

In the United States, the potential distribution range of EBR under historical climate conditions is larger than that of ESC, with 66.16% and 58.57% of the total land area or 6.19 × 10^6^ km^2^ and 5.48 × 10^6^ km^2^, respectively (Table 1).

**Figure 5:**
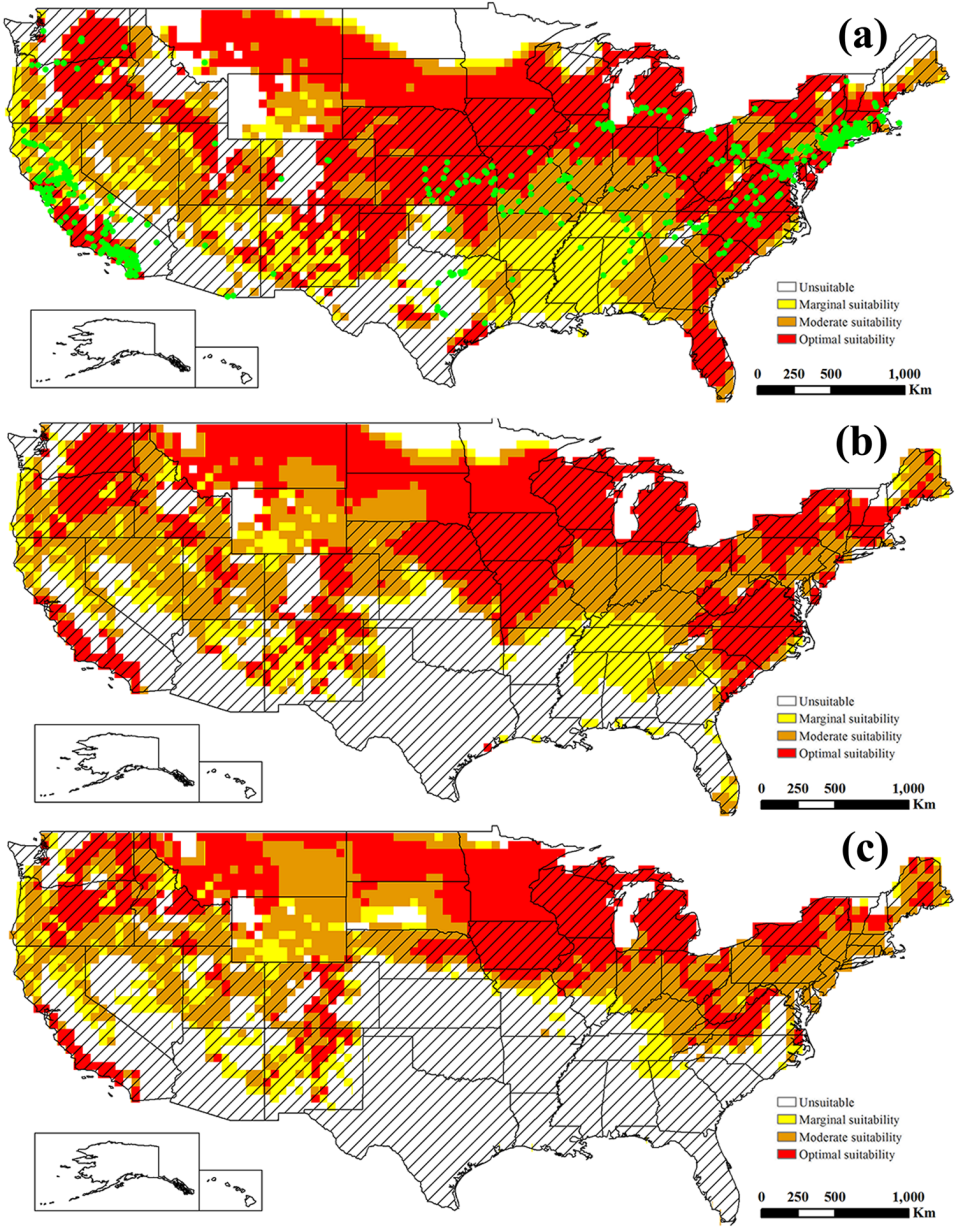
The potential distributions for ESC in the United States, (**a**) under the historical climate, (**b**) under the future climate change scenario RCP 8.5 by the middle of 21st century, (**c**) under the future climate change scenario RCP 8.5 by the end of 21st century. Slash indicate the distribution areas of tree-of-heaven. Green points indicate the distribution locations of tree-of-heaven. White indicates unsuitable areas (EI = 0); Yellow indicates areas of marginal suitability (0 < EI ≤ 10); Orange indicates areas of moderate suitability (10 < EI ≤ 20); Red indicates areas of optimal suitability (EI ≥ 20). The map in this figure was generated by ArcGIS 10.1 (ESRI, Redlands, CA, USA, http://resources.arcgis.com/en/home/).

**Figure 6:**
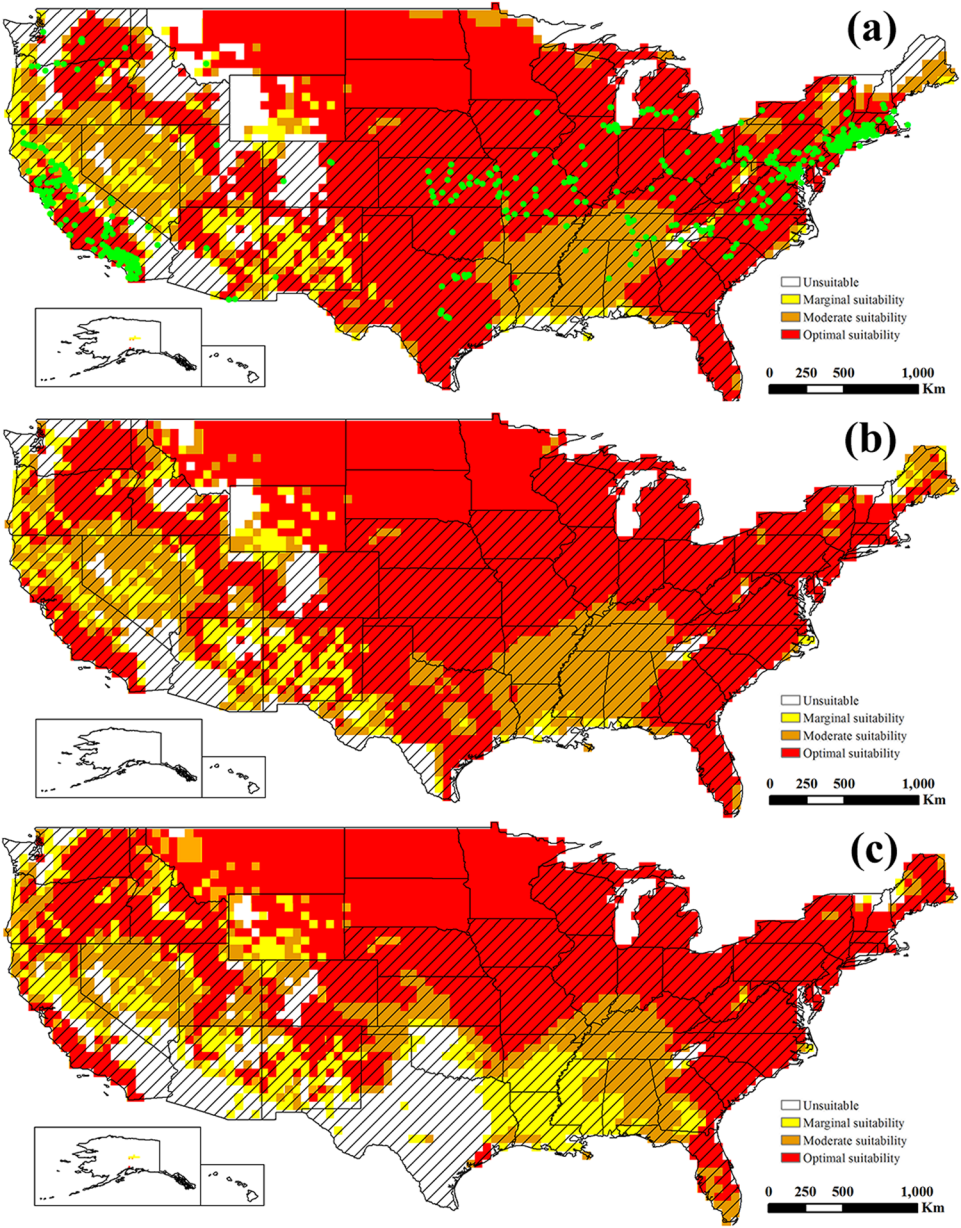
The potential distributions for EBR in the United States, (**a**) under the historical climate, (**b**) under the future climate change scenario RCP 8.5 by the middle of 21st century, (**c**) under the future climate change scenario RCP 8.5 by the end of 21st century. Slash indicate the distribution areas of tree-of-heaven. Green points indicate the distribution locations of tree-of-heaven. White indicates unsuitable areas (EI = 0); Yellow indicates areas of marginal suitability (0 < EI ≤ 10); Orange indicates areas of moderate suitability (10 < EI ≤ 20); Red indicates areas of optimal suitability (EI ≥ 20). The map in this figure was generated by ArcGIS 10.1 (ESRI, Redlands, CA, USA, http://resources.arcgis.com/en/home/).

The following areas were projected to be optimal suitability for ESC, which included primarily all the east coast states of the United States, the coast of California, Montana, South Dakota, Nebraska, Kansas, Oklahoma, New Mexico, northern Texas, southern Minnesota, Iowa, Missouri, Wisconsin, Michigan, Illinois, Indiana, Ohio, Kentucky, West Virginia, and Pennsylvania. The areas of moderate suitability for ESC, included Washington, Oregon, northern California, Idaho, Nevada, Utah, Arizona, Arkansas, Tennessee, and Alabama. Narrow geographical zones in northern Minnesota and North Dakota, Louisiana and Mississippi were projected to be areas of marginal suitability for ESC (Fig. 5a). For EBR, areas of optimal suitability are almost covered by the optimal suitability areas of ESC, but Texas, North Dakota, Minnesota, Missouri, southern Illinois, southern Indiana and southern Kentucky need to be added as optimal suitability areas for EBR. Areas of moderate suitability for EBR were concentrated in Alabama, Mississippi, Arkansas, Louisiana, Tennessee and Nevada. Areas of marginal suitability for EBR were projected in the central of New Mexico, northern California and Arizona (Fig. 6a). Additionally, 96.69 percent or 1022 of distribution points of tree-of-heaven were located in the projected potential distributions of ESC, and 96.88 percent or 1024 distribution points of tree-of-heaven for that of EBR in the United States.

Compared with areas of potential distribution under the historical climate, the potential distribution range of ESC decreased from 5.48 × 10^6^ km^2^ to 4.94 × 10^6^ km^2^ by the middle of 21st century, 4.10 × 10^6^ km^2^ by the end of 21st century (Table 2), which included areas that became unsuitable in Texas, Louisiana, Alabama, Georgia, Mississippi, Florida, South Carolina, Kansas, Oklahoma (Fig. 5b,c). Areas of potential distribution for EBR showed a slight increase to 6.42 × 10^6^ km^2^ by the middle of 21st century, a slight decrease to 6.07 × 10^6^ km^2^ by the end of 21st century (Table 2) under the AR5 climate change scenario RCP 8.5. The changes of potential distributions primarily involved areas in western Montana and Wyoming, central Colorado, Idaho that changed from unsuitable to suitable, and Texas, Oklahoma changed unsuitable (Fig. 6b,c).

**Table 2:** The climatically suitable areas (EI > 0) in the United States for ESC and EBR under the historical and future climate change scenario RCP 8.5.

Scenarios		ESC		EBR	
		Total area (10^6^ km^2^)	% Total land area	Total area (10^6^ km^2^)	% Total land area
Historical climate	EI = 0	3.88	41.43	3.17	33.84
	0 < EI ≤ 10	1.00	10.63	0.52	5.51
	10 < EI ≤ 20	1.58	16.91	1.18	12.61
	20 < EI ≤ 100	2.91	31.03	4.50	48.03
2046–2065 RCP 8.5	EI = 0	4.42	47.21	2.95	31.47
	0 < EI ≤ 10	0.84	8.96	0.59	6.31
	10 < EI ≤ 20	1.72	18.40	1.29	13.80
	20 < EI ≤ 100	2.38	25.43	4.53	48.42
2081–2100 RCP 8.5	EI = 0	5.26	56.17	3.30	35.19
	0 < EI ≤ 10	0.58	6.22	0.94	9.99
	10 < EI ≤ 20	1.70	18.14	1.16	12.39
	20 < EI ≤ 100	1.82	19.47	3.97	42.44

## Discussion

*Ailanthus altissima* is a notable example of a species that became invasive outside its natural climate zone; the species is native to subtropical/warm temperate climates but invades climates ranging from cool temperate to tropical^2, 45–47^ (Table S1). Although *A. altissima* is clearly widely adaptable to different climates and widely distributed, many trees of *A. altissima* die from the attack of two weevils, ESC and EBR in China. Therefore, two weevils can be introduced into these areas to agaist *A. altissima*. However, whether the climate can reach a satisfactory level for growth of ESC and EBR is an essential prerequisite to introduce the two weevils into the distribution areas of *A. altissima*. Therefore, the areas with suitable climate for ESC and EBR under historical climate and a future climate change scenario RCP 8.5 were modeled using CLIMEX 4.0 in this study.

The results indicate that both ESC and EBR are expected to be able to establish itself in the regions where tree-of-heaven have been planted. Nomatter what historical climate or a future climate, the projected potential distributions of ESC and EBR included almost all actual current areas of distribution for tree-of-heaven. The use of irrigation scenarios has an important impact on the distribution of ESC and EBR in arid areas, such as northwest China. We propose the change trend of ESC and EBR distribution areas under climate change scenario RCP 8.5 by the middle and latter of 21st century. The results of this study provide an important basis for the introduction of ESC and EBR as potential biological control agents against tree-of-heaven. Additionally, notable, there is a special problem about the expression of the scientific name of ESC need to be explained. Most of the research articles have documented that the scientific name of this weevil species is *Eucryptorrhynchus chinensis* (Olivier), 1790^1, 12, 14, 15^, however, the correct scientific name should be *Eucryptorrhynchus scrobiculatus* Motschulsky, 1854 on basis of the monographs of “A World Catalogue of Families and Genera of Cruculionoidea”^48^ and “Catalogue of Palaearctic Coleptera”^49^. Thus, in fact, the weevil of ESC and *Eucryptorrhynchus chinensis* are the same species.

Although we have greatly increased confidence in the introduction of ESC and EBR as potential biological control agents against tree-of-heaven, there are still many issues to be studied in determining the two weevils as biological control agents. The determination of biological control agents to pests requires many complex studies, such as host species, generation development, natural enemies, climate suitability, contributions to ecosystem services and other non-target effects of biological control agents in the introduced and invaded areas^50–52^. For the species ESC and EBR in this study are the most destructive pests of *Ailanthus altissima* because of the climate suitability for survival, adequate food resources of tree-of-heaven in China. However, the possible mechanism is not clear of introducing EBR or ESC to limit *A. altissima* populations in the introduced and invaded areas. Kok *et al*.^10^ evaluated EBR for potential as a biological control agent in the United States, and the results of development and rearing of EBR indicated that the weevil could complete its life cycle in a cut tree of heaven log in the laboratory, and the adult and larvae feeding test indicated a high level of specificity for tree-of-heaven. With the results of projecting the potential distribution areas of EBR in this study (Figs 2 and 6), therefore, we can only tentatively assume that EBR is the biological control agent of tree-of-heaven. As for ESC, Although the potential areas of suitability have been projected (Figs 1 and 5), there are no researches on the generation development and host specificity. Therefore, further studies are needed to determine whether ESC can be used as a biological control agent against tree-of-heaven.

In this study, we only considered a single factor of climate suitability for ESC and EBR, because it is important whether the two weevils could survive in the distribution areas of *Ailanthus altissima* as potential biological control agents to agaist *A. altissima*. Considering only climate suitability, both ESC and EBR can be introduced into the distribution areas of *A. altissima* as potential biological control agents under historical climate and future climate change scenario RCP 8.5 in the middle and latter of 21st century. As greenhouse gas emissions continue to increase, the resulting global warming will have a tremendous effect on insect distributions^53^. With the limitations of cold stress overcome, the areas of distribution will extend to the north. As the results of this study showed, the areas of potential distribution for both ESC and EBR extended into northern Canada in North America and Finland in Europe under the climate change scenario RCP 8.5 in the middle and latter of the 21st century. Meanwhile, with global warming, the areas of potential distribution were projected to decrease in the Southern Hemisphere, with this reduction likely caused by increased effects of thermal stress as temperatures increased. Furthermore, according to the current areas of distribution of ESC and EBR in China, we can found that the distribution data for these species includes xeric areas in western China. In these areas, irrigation is used to protect the normal growth of plants in farmland and forest shelter belt^54, 55^. Therefore, based on areas across the globe considered to be under irrigation according to Siebert *et al*.^44^, the irrigation scenarios of 2.5 mm day^−1^ in the summer and 1.5 mm day^−1^ in the winter are used as a top-up to natural rainfall in this study. If irrigation scenarios are not used, these areas will be unsuitable areas where they are actually suitable areas because of the effects of dry stress, such as northwestern China, western United States, southern Australia, southern South America, southern Africa and Western Asia. The projected potential distribution areas of ESC and EBR are consistent with the current distribution of tree-of-heaven, except Southeast Asia for wet stress.

In addition, Zhang^56^ projected the distribution of EBR in the United States under the historical climate using CLIMEX 1.1 and showed areas of potential distribution in approximately twenty states in the central and western United States. The projections of this earlier version of the model differed greatly from the results of the CLIMEX 4.0 model used in this study. However, the earlier model most likely caused errors in projection because of the poor fit to the current distribution records for China, particularly in Jiangsu, Shanghai, Hubei and Hunan. Moreover, there is also difference in climate data used in projection, 67419 climate stations data around the world in this study and larger than 2031 climate stations in earlier projection of Zhang, which may also have a very important impact on projection. A single number EI value is provided to describe how favourable the climate of a location is for a particular species, and this figure can be broken down into component parts for a more detailed examination of the species’ response to climate at any given location^35^. The EI is scaled between 0 and 100, with an EI close to 0 indicating that the location is not favourable for the long-term survival of the species. EI values of 100 are only achievable under constant and ideal conditions comparable in incubators^35–38, 57^. Watt *et al*.^40^ projected the future distribution of *Melaleuca quinquenervia* using CLIMEX with EI value of greater or equal to 20 as optimal suitable match; Park *et al*.^58^ projected the potential geographic distribution of *Thrips palmithe* with EI value of greater or equal to 25 as optimal suitable match; Kumar *et al*.^41^ assessed the global risk of establishment of *Cydia pomonella* with EI value of greater to 10 as highly suitable match. In this study, average EI values of ESC and EBR are 36.5 and 32.8 in Ningxia Hui Autonomous Region with serious damage to *A. altissima*. Average EI values of ESC and EBR are 21.3 and 24.8 in Shandong province in China with relatively serious damage to *Ailanthus altissima*. Average EI value of ESC is 0.9 in Fujian province in China with only one damage report, and average EI value of EBR is 3.5 in Heilongjiang province in China with only two damage reports. Therefore, the assumptions of a suitable match of EI value used in this study are scientific and reliable.

The CLIMEX model is based partly on the assumption that if you know where a species lives you can infer what climatic conditions it can tolerate^35–38^. This is the cardinal assumption underpinning most species distribution models. However, where other models such as Bioclim, Domain, GARP and MaxEnt attempt to characterise the environment occupied by the species, CLIMEX simulates the mechanisms that limit species’ geographical distributions and determine their seasonal phenology, and to some extent their relative abundance^57^. Where most models focus on describing the relationship between the occurrences of the species with respect to static environmental covariates, CLIMEX describes how the species responds to climatic variables at appropriate temporal scales (daily or weekly). In CLIMEX model, the actual geographical distribution and biological parameters are used to determine the final projection parameters, which can reduce the deviation due to only rely on climate^36, 37^. In addition, we can have more confidence in these results if they have been carefully validated using independent (geographically biased) data. However, ESC and EBR currently have not spread to other areas; therefore, biological and ecological data were based only on native distribution data to set initial CLIMEX parameters. The final values of parameters in this study were established by calibrating parameter values until the simulated geographical distribution coincided as closely as possible with the observed distribution in China. Moreover, the effects of competition among species, natural enemies and hosts on the projection of potential distribution areas have not been adequately considered in this study.

Although this study presents a good result, there are still many problems. Climate is not the only condition for the projection of areas of potential distribution; dispersal and species interactions, such as host availability, competition, and the effect of natural enemies, must also be considered^59^. Moreover, limiting factors, such as human activities, soils, and mountains and other geographical barriers, should be considered because they also affect the projections for potential areas of distribution. The method of CLIMEX modelled mainly considers climatic factor, regardless of the above factors in this study, and the predictions based on the model and the results discussed above surely have some limitations. Therefore, these additional factors should also be considered, and more trials and detailed risk assessments need to be studied to determine whether ESC and EBR can be used as biological control agents against tree-of-heaven.

## Methods

### CLIMEX model

CLIMEX 4.0 (Hearne Scientific Software, Melbourne, Australia) was used to estimate the climatic suitability for ESC and EBR worldwide. The potential areas of suitability and relative abundance of the two species were projected in CLIMEX from known geographical distributions and some laboratory data, including developmental threshold temperatures, developmental optimum temperatures, and soil moisture that were used to fit or fine-tune CLIMEX parameter values^38, 43^. Two functions, Compare Locations and Match Climates, can be used to project potential areas of suitability for these weevils. Compared with Match Climates, Compare Locations uses both the current areas of distribution and the biological data. Therefore, Compare Locations was used in this study. Additionally, the Climate Change Scenario option in the Compare Locations model was used to adjust climate change parameters. A series of annual indices were used in the CLIMEX model that integrate the weekly responses of a population to climate^38^. Annual Growth Index was used to describe the potential population growth during favorable conditions, and eight Stress Indices, including cold, wet, hot, dry, cold-wet, cold-dry, hot-wet, and hot-dry, were used to determine the probability that the population could survive unfavorable conditions. The Growth and Stress Indices were combined into an Ecoclimatic Index (EI) to give an overview of the climatic suitability of a target location for a species. The range of EI is scaled from 0 for locations at which a species is not able to persist to 100 for locations that are optimal for a species. However, an EI of more than 30 represents a climate that is actually very favorable for a species^38^. In this study, commonly used criteria were used to classify the EI values: EI = 0, area unsuitable; 0 < EI ≤ 10, area marginally suitable; 10 < EI ≤ 20, area moderately suitable; and EI ≥ 20, area optimal.

### Distribution of ESC and EBR

**Figure 7:**
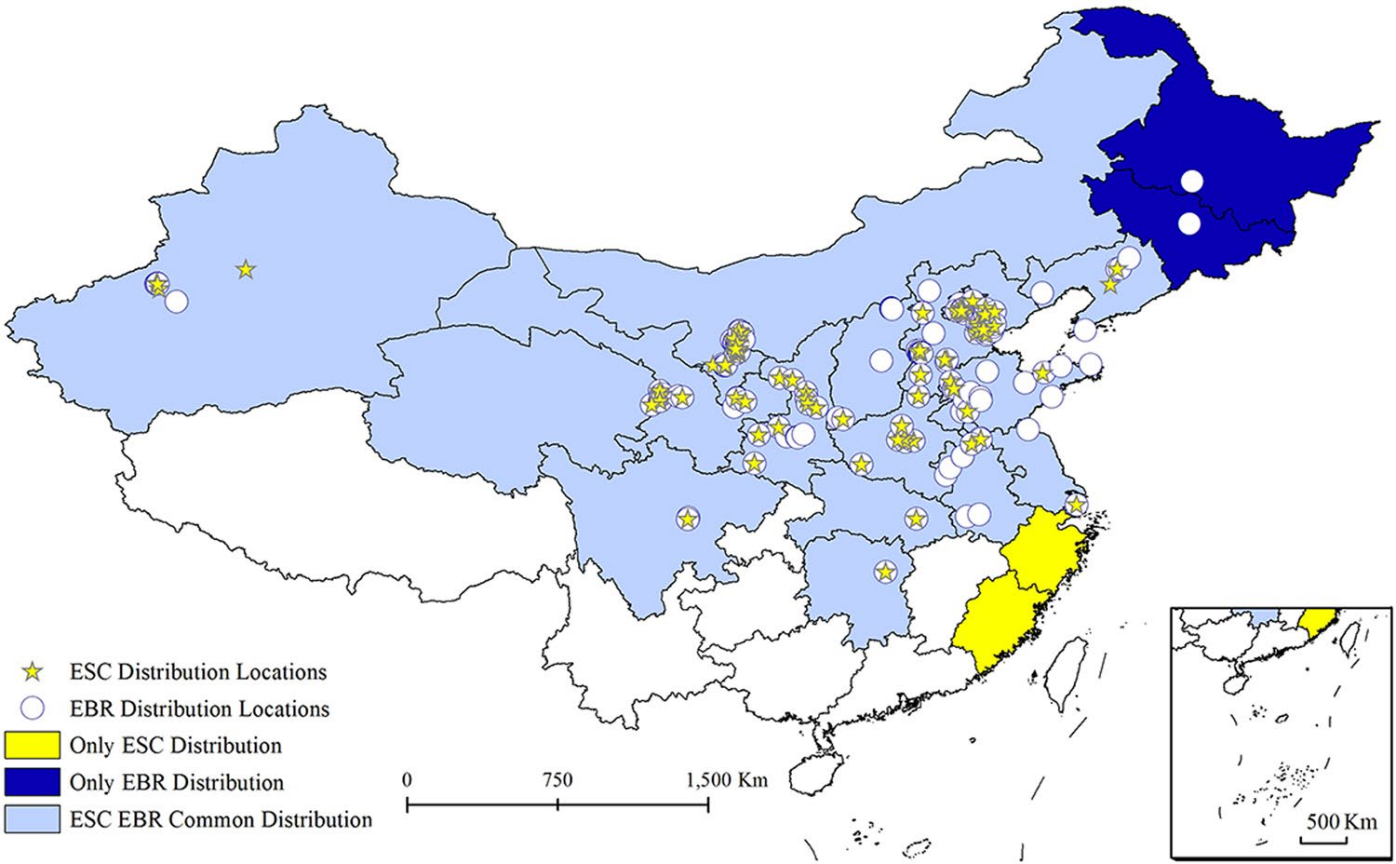
The current distributions of ESC and EBR in China. The map in this figure was generated by ArcGIS 10.1 (ESRI, Redlands, CA, USA, http://resources.arcgis.com/en/home/).

The genus *Eucryptorrhynchus* Heller 1937 includes the two species ESC and EBR in the Palearctic region^48, 49^, which are both native to China. The distribution of ESC is limited in China from Liaoning to Sichuan in approximately 21 provincial regions^11, 14^, whereas EBR is distributed in China (21 provincial regions: Heilongjiang, Jilin, Liaoning, Inner Mongolia, Beijing, Tianjin, Hebei, Shanxi, Shandong, Henan, Shaanxi, Ningxia, Gansu, Qinghai, Xinjiang, Jiangsu, Shanghai, Anhui, Hubei, Hunan, and Sichuan) and also in North Korea^11, 12^. Additionally, EBR may have been introduced into the United States by quarantine evaluation as a biological agent against *A. altissima* ^10, 17^. The known occurrences of ESC and EBR were determined from published literature (Fig. 7 and Tables S2 and S3).

### Historical climate data and climate change scenarios

The CliMond 10′ resolution (approximately 1 km) climate data (download from https://www.climond.org/ClimateData.aspx) were used to represent the historical climate (averaging period 1961–1990) in CLIMEX 4.0, which consisted of five climatic variables, including average minimum monthly temperature (T_min_), average maximum monthly temperature (T_max_), average monthly precipitation (P_total_), and relative humidity at 09: 00 h (RH 09: 00) and 15: 00 h (RH 15: 00)^38^. As for future climate, the scenario RCP 8.5 is considered for the projection of the potential distribution of ESC and EBR in the middle and latter 21st century. The scenario RCP 8.5 is considered the most pessimistic for the 21st century in terms of greenhouse gas emissions, being consistent with no policy change to reduce emissions, rapid increase in methane emissions and heavy reliance on fossil fuels^60^. What scant evidence we have from present emissions trajectories and historical patterns of behaviour suggests that the RCP 8.5 should be considered the business as usual scenario, and perhaps the most likely. Compared with the 1986–2005 climate data, the global temperatures and rainfall levels are projected to increase 1.4–2.6 °C and 1.4–7.8% by the middle 21st century (2046–2065), and 2.6–4.8 °C and 2.6–14.4% by the end of 21st century (2081–2100), based on Dong and Gao^61^, Zhao *et al*.^62^, Qin *et al*.^26^, and IPCC AR5^25^.

### Establishment of CLIMEX model parameters

CLIMEX parameters for ESC and EBR were manually and iteratively adjusted until the simulated suitability patterns estimated by the EI values most closely matched the known geographical distribution and the abundance throughout the range. Parameter values for ESC and EBR are presented in Table 3. The biology of ESC and EBR has been investigated in many studies since the 1980s; therefore, the relevant biological data were chosen directly to specify the parameters of CLIMEX. The detailed biological characterizations of ESC and EBR were provided by the in-depth studies of Yu^16^, Yang *et al*.^63^, Zhang^56^, and Ge^12^ and included the effects of temperature and moisture on development as references to specify parameters.

**Table 3:** CLIMEX parameter values for ESC and EBR.

CLIMEX–parameter	ESC value	EBR value
DV0–Lower threshold temperature	5.95 °C	6.7 °C
DV1–Lower optimum temperature	18 °C	20 °C
DV2–Upper optimum temperature	22 °C	24 °C
DV3–Upper threshold temperature	32 °C	36 °C
PDD–Degree-day threshold	1332 °C Days	1350 °C Days
SM0–Lower soil moisture threshold	0.1	0.1
SM1–Lower optimum soil threshold	0.3	0.3
SM2–Upper optimum soil threshold	0.9	0.9
SM3–Upper soil moisture threshold	1.2	1.2
TTCS–Cold stress temperature threshold	−17 °C	−19 °C
THCS–Cold stress accumulation rate	−0.05 Week^−1^	−0.003 Week^−1^
TTHS–heat stress temperature threshold	34	36
THHS–heat stress accumulation rate	0.045 Week^−1^	0.01 Week^−1^
SMDS–Dry stress soil moisture threshold	0.1	0.1
HDS–Dry stress accumulation rate	−0.01 Week^−1^	−0.018 Week^−1^
SMWS–Wet stress soil moisture threshold	1.2	1.2
HWS–Wet stress accumulation rate	0.011 Week^−1^	0.011 Week^−1^

CLIMEX model parameter values for ESC and EBR were primarily derived from biological and ecological data in China. Biological and ecological data of ESC showed that the minimum developmental temperature (DV0) was 5.95 °C, and because the larvae grew very slowly and the growth period lengthened, the upper temperature threshold (DV3) appeared to be 32 °C. The degree-day (DD) accumulation requirement for survival was approximately 1332 DD^14, 63^. The suitable range of soil moisture for oviposition was from 5% to 15%^64^, with strong tolerance to high soil moisture by adults. According to the known distribution data, the northern distribution boundary was in Shenyang, Liaoning Province (123.31°N, 41.8°E), the western boundary in Aksu, Xinjiang Autonomous Region (80.26°N, 41.17°E), and the southern boundary in Sichuan Province^11, 16, 56^. Therefore, the stress parameters were set at cold stress temperature threshold (TTCS) = −17 °C, heat stress temperature threshold (TTHS) = 34 °C, dry stress soil moisture threshold (SMDS) = lower soil moisture threshold (SM0) = 0.1, and wet stress soil moisture threshold (SMWS) = upper soil moisture threshold (SM3) = 1.2. Similar to ESC, biological and ecological data for EBR were obtained from published articles. According to the detailed research by Zhang^56^, the DV0 was 6.7 °C, and the DV3 appeared to be 36 °C. The DD accumulation requirement for survival was approximately 1352 DD. The adults have strong cold resistance and survived at −17 °C for 6 h, and TTCS = −19 °C. Based on different experimental sets from 20 °C to 32 °C, relative humidity from 41% to 92% was suitable for the development of eggs, and lower optimum temperature (DV1) = 20 °C, upper optimum temperature (DV2) = 24 °C. Additionally, we apply an irrigation scenario of 2.5 mm day^−1^ in the summer and 1.5 mm day^−1^ in the winter as top-up to project the potential distribution of ESC and EBR. We use the irrigation areas identified by Siebert *et al*.^44^ to produce a composite map, comprising both irrigated and non-irrigated areas, to show the overall projected suitability for ESC and EBR.

### Generating a map of potential distribution areas

Maps of potential distribution areas were generated using ArcGIS 10.1 software (http://resources.arcgis.com/en/home/) based on EI values from the CLIMEX model. For a more intuitive interpretation of the results, ArcGIS 10.1 was used to transformation the data. The point to raster tool in ArcGIS was used to create a result surface, and the thematic mapping function was used to map the surface to show the areas of different climatic suitability for ESC and EBR globally.

## Electronic supplementary material

Supplementary Information
