# Supplementary material for: Projecting potential distribution of Eucryptorrhynchus scrobiculatus Motschulsky and E. brandti (Harold) under historical climate and RCP 8.5 scenario
Source: Sci Rep. 2017 Aug 22;7:9163. doi: 10.1038/s41598-017-09659-3 (PMC5567332; doi:10.1038/s41598-017-09659-3)
Supplement: Supplementary file 1 — Supplementary Information [file 41598_2017_9659_MOESM1_ESM.doc]

**Projecting potential distribution of *Eucryptorrhynchus scrobiculatus* Motschulsky and *E. brandti* (Harold) under historical climate and RCP 8.5 scenario**

**Yingchao Ji, Wen Luo, Ganyu Zhang & Junbao Wen***

Summary of global distribution of *Ailanthus altissima*, and summary of detailed distribution sites of ESC and EBR used for the potential distributions prediction. Table S1 provides a summary of global distribution by countries of *Ailanthus altissima*; Table S2 provides a summary of the detailed distribution sites of ESC; Table S3 provides a summary of the detailed distribution sites of EBR.

***Table S1: Summary of global distribution by countries of Ailanthus altissima***

| **Country** | **Distribution** | **Origin** | **Invasive** | **Nature** | **References** |
| --- | --- | --- | --- | --- | --- |
| **Asia** | | | | | |
| [China](http://www.cabi.org/isc/datasheet/108398) | Widespread | Native |  |  | [Li, 1963](http://www.cabi.org/isc/datasheet/3889#20057012467); [Zheng, 1978](http://www.cabi.org/isc/datasheet/3889#20057024528); [EPPO, 2014](http://www.cabi.org/isc/datasheet/3889#20127201272); Liu, 1988 |
| [Georgia](http://www.cabi.org/isc/datasheet/108433) | Present |  |  |  | [EPPO, 2014](http://www.cabi.org/isc/datasheet/3889#20127201272) |
| [India](http://www.cabi.org/isc/datasheet/108459) | Present | Introduced |  |  | [Singh](http://www.cabi.org/isc/datasheet/3889#19930668446) *et al.*, 1992; [EPPO, 2014](http://www.cabi.org/isc/datasheet/3889#20127201272) |
| [Indonesia](http://www.cabi.org/isc/datasheet/108455) | Present | Introduced |  |  | [EPPO, 2014](http://www.cabi.org/isc/datasheet/3889#20127201272) |
| [Iran](http://www.cabi.org/isc/datasheet/108462) | Present | Introduced |  | Planted | [Luna, 1996](http://www.cabi.org/isc/datasheet/3889#19960603784); [EPPO, 2014](http://www.cabi.org/isc/datasheet/3889#20127201272) |
| [Israel](http://www.cabi.org/isc/datasheet/108457) | Widespread | Introduced |  |  | [EPPO, 2014](http://www.cabi.org/isc/datasheet/3889#20127201272) |
| [Japan](http://www.cabi.org/isc/datasheet/108467) | Present |  | Invasive | Natural | [Singh](http://www.cabi.org/isc/datasheet/3889#19930668446) *et al.*, 1992; [EPPO, 2014](http://www.cabi.org/isc/datasheet/3889#20127201272) |
| [Korea](http://www.cabi.org/isc/datasheet/108477) | Present | Introduced |  | Planted | [EPPO, 2014](http://www.cabi.org/isc/datasheet/3889#20127201272) |
| [Malaysia](http://www.cabi.org/isc/datasheet/108514) | Present |  |  |  | [EPPO, 2014](http://www.cabi.org/isc/datasheet/3889#20127201272) |
| [Pakistan](http://www.cabi.org/isc/datasheet/108537) | Present | Introduced | Invasive | Planted | [Hussain, 2002](http://www.cabi.org/isc/datasheet/3889#20057050401); [EPPO, 2014](http://www.cabi.org/isc/datasheet/3889#20127201272) |
| [Turkey](http://www.cabi.org/isc/datasheet/108587) | Present | Introduced |  |  | [EPPO, 2014](http://www.cabi.org/isc/datasheet/3889#20127201272) |
| **Africa** | | | | | |
| Algeria | Present |  |  |  | EPPO, 2014 |
| Libya |  |  |  |  | EPPO, 2014 |
| Morocco |  |  |  |  | EPPO, 2014 |
| South Africa | Present | Introduced | Invasive |  | Henderson, 2001; EPPO, 2014 |
| **North America** | | | | | |
| [Canada](http://www.cabi.org/isc/datasheet/108388) | Restricted distribution | Introduced | Invasive |  | [Weber, 2003](http://www.cabi.org/isc/datasheet/3889#20033158536); [EPPO, 2014](http://www.cabi.org/isc/datasheet/3889#20127201272) |
| [Mexico](http://www.cabi.org/isc/datasheet/108513) | Present | Introduced |  |  | [Weber, 2003](http://www.cabi.org/isc/datasheet/3889#20033158536); [EPPO, 2014](http://www.cabi.org/isc/datasheet/3889#20127201272) |
| [USA](http://www.cabi.org/isc/datasheet/108597) | Widespread | Introduced | Invasive |  | [Feret, 1985](http://www.cabi.org/isc/datasheet/3889#19860607318); [Shah, 1997](http://www.cabi.org/isc/datasheet/3889#19980305927); [Holm](http://www.cabi.org/isc/datasheet/3889#20057025044) *et al.*, 1979; [EPPO, 2014](http://www.cabi.org/isc/datasheet/3889#20127201272) |
| **South America** | | | | | |
| [Argentina](http://www.cabi.org/isc/datasheet/108359) | Present | Introduced |  |  | [Weber, 2003](http://www.cabi.org/isc/datasheet/3889#20033158536); [EPPO, 2014](http://www.cabi.org/isc/datasheet/3889#20127201272) |
| [Chile](http://www.cabi.org/isc/datasheet/108396) | Present | Introduced |  |  | [Weber, 2003](http://www.cabi.org/isc/datasheet/3889#20033158536); [Holm](http://www.cabi.org/isc/datasheet/3889#20057025044) *et al.*, 1979 |
| **Europe** | | | | | |
| [Albania](http://www.cabi.org/isc/datasheet/108354) | Present |  |  |  | [EPPO, 2014](http://www.cabi.org/isc/datasheet/3889#20127201272) |
| [Austria](http://www.cabi.org/isc/datasheet/108361) | Present |  |  |  | [EPPO, 2014](http://www.cabi.org/isc/datasheet/3889#20127201272) |
| [Belgium](http://www.cabi.org/isc/datasheet/108370) | Present |  |  |  | [EPPO, 2014](http://www.cabi.org/isc/datasheet/3889#20127201272) |
| [Czech Republic](http://www.cabi.org/isc/datasheet/108409) | Widespread | Introduced | Invasive |  | [DAISIE, 2014](http://www.cabi.org/isc/datasheet/3889#20127200434); [EPPO, 2014](http://www.cabi.org/isc/datasheet/3889#20127201272) |
| [France](http://www.cabi.org/isc/datasheet/108429) | Present | Introduced | Invasive |  | [Kowarik, 1983](http://www.cabi.org/isc/datasheet/3889#19840697297); [Cronk & Fuller, 1995](http://www.cabi.org/isc/datasheet/3889#19952306429); [EPPO, 2014](http://www.cabi.org/isc/datasheet/3889#20127201272) |
| [Germany](http://www.cabi.org/isc/datasheet/108410) | Present | Introduced | Invasive |  | [DAISIE, 2014](http://www.cabi.org/isc/datasheet/3889#20127200434); [EPPO, 2014](http://www.cabi.org/isc/datasheet/3889#20127201272) |
| [Greece](http://www.cabi.org/isc/datasheet/108443) | Present | Introduced | Invasive |  | [Cronk & Fuller, 1995](http://www.cabi.org/isc/datasheet/3889#19952306429); [EPPO, 2014](http://www.cabi.org/isc/datasheet/3889#20127201272) |
| [Hungary](http://www.cabi.org/isc/datasheet/108454) | Present | Introduced | Invasive |  | [Cronk & Fuller, 1995](http://www.cabi.org/isc/datasheet/3889#19952306429); [EPPO, 2014](http://www.cabi.org/isc/datasheet/3889#20127201272) |
| [Italy](http://www.cabi.org/isc/datasheet/108464) | Present | Introduced | Invasive |  | [Badalamenti](http://www.cabi.org/isc/datasheet/3889#20127200955) *et al.*, 2012; [EPPO, 2014](http://www.cabi.org/isc/datasheet/3889#20127201272) |
| [Malta](http://www.cabi.org/isc/datasheet/108509) | Present | Introduced | Invasive |  | [DAISIE, 2014](http://www.cabi.org/isc/datasheet/3889#20127200434); [EPPO, 2014](http://www.cabi.org/isc/datasheet/3889#20127201272) |
| [Moldova](http://www.cabi.org/isc/datasheet/108495) | Present | Introduced | Invasive |  | [DAISIE, 2014](http://www.cabi.org/isc/datasheet/3889#20127200434); [EPPO, 2014](http://www.cabi.org/isc/datasheet/3889#20127201272) |
| [Netherlands](http://www.cabi.org/isc/datasheet/108522) | Present |  |  |  | [EPPO, 2014](http://www.cabi.org/isc/datasheet/3889#20127201272) |
| [Portugal](http://www.cabi.org/isc/datasheet/108542) | Present | Introduced | Invasive |  | [DAISIE, 2014](http://www.cabi.org/isc/datasheet/3889#20127200434); [EPPO, 2014](http://www.cabi.org/isc/datasheet/3889#20127201272) |
| [Romania](http://www.cabi.org/isc/datasheet/108548) | Present | Introduced | Invasive |  | [DAISIE, 2014](http://www.cabi.org/isc/datasheet/3889#20127200434); [EPPO, 2014](http://www.cabi.org/isc/datasheet/3889#20127201272) |
| [Serbia](http://www.cabi.org/isc/datasheet/108549) | Present |  |  |  | [EPPO, 2014](http://www.cabi.org/isc/datasheet/3889#20127201272) |
| [Slovenia](http://www.cabi.org/isc/datasheet/108559) | Present |  |  |  | [EPPO, 2014](http://www.cabi.org/isc/datasheet/3889#20127201272) |
| [Spain](http://www.cabi.org/isc/datasheet/108421) | Widespread | Introduced | Invasive |  | [DAISIE, 2014](http://www.cabi.org/isc/datasheet/3889#20127200434); [Sanz-Elorza](http://www.cabi.org/isc/datasheet/3889#20077201870) *et al.*, 2004; [EPPO, 2014](http://www.cabi.org/isc/datasheet/3889#20127201272) |
| [Switzerland](http://www.cabi.org/isc/datasheet/108393) | Present |  |  |  | [EPPO, 2014](http://www.cabi.org/isc/datasheet/3889#20127201272) |
| [UK](http://www.cabi.org/isc/datasheet/108431) | Present | Introduced |  |  | [Weber, 2003](http://www.cabi.org/isc/datasheet/3889#20033158536); [Hu, 1979](http://www.cabi.org/isc/datasheet/3889#19790376426); [EPPO, 2014](http://www.cabi.org/isc/datasheet/3889#20127201272) |
| [Ukraine](http://www.cabi.org/isc/datasheet/108592) | Present | Introduced | Invasive |  | [DAISIE, 2014](http://www.cabi.org/isc/datasheet/3889#20127200434); [EPPO, 2014](http://www.cabi.org/isc/datasheet/3889#20127201272) |
| **Oceania** | | | | | |
| [Australia](http://www.cabi.org/isc/datasheet/108362) | Present | Introduced | Invasive |  | [Cronk & Fuller, 1995](http://www.cabi.org/isc/datasheet/3889#19952306429); [Holm](http://www.cabi.org/isc/datasheet/3889#20057025044) *et al.*, 1979; [EPPO, 2014](http://www.cabi.org/isc/datasheet/3889#20127201272) |
| [New Zealand](http://www.cabi.org/isc/datasheet/108528) | Present | Introduced |  |  | [Weber, 2003](http://www.cabi.org/isc/datasheet/3889#20033158536); [EPPO, 2014](http://www.cabi.org/isc/datasheet/3889#20127201272) |

**Table S2: Summary of the detailed distribution sites of ESC used to the potential distributions prediction.**

| **Country** | **Locations** | **Longitude** | **Latitude** | **References** |
| --- | --- | --- | --- | --- |
| China | Akesu, Xinjiang autonomous region | 80.26 | 41.17 | Zhou et al., 2007 |
| China | Akesu, Xinjiang autonomous region | 80.27 | 41.17 |  |
| China | Akesu, Xinjiang autonomous region | 80.38 | 40.93 |  |
| China | Akesu, Xinjiang autonomous region | 80.27 | 41.16 |  |
| China | Akesu, Xinjiang autonomous region | 80.3 | 41.14 |  |
| China | Anshan, Liaoning province | 122.99 | 41.11 | Xu et al., 2015 |
| China | Anyang, Henan province | 114.39 | 36.1 | Qin, 1996 |
| China | Baoji, Shaanxi province | 107.24 | 34.36 | Wang et al., 2008 |
| China | Baoshan reservation park, Henan province | 111.84 | 33.05 | Cao et al., 2012 |
| China | Beijing | 116.4 | 39.9 | Zhang et al., 2014 |
| China | Beijing | 116.83 | 40.37 |  |
| China | Beijing | 116.22 | 39.9 |  |
| China | Beijing | 116.48 | 39.8 |  |
| China | Beijing | 116.29 | 40.04 |  |
| China | Beijing | 116.39 | 40.02 |  |
| China | Beijing | 116.1 | 39.94 |  |
| China | Beijing | 116.34 | 40.01 |  |
| China | Beijing | 116.32 | 39.94 |  |
| China | Changsha, Hunan province | 112.94 | 28.23 | Zhang et al., 2009 |
| China | Fu county, Shaanxi province | 109.43 | 35.76 | Xiao et al., 2008 |
| China | Ganquan county, Shaanxi province | 109.37 | 35.99 | Xiao et al., 2008 |
| China | Guyuan, Ningxia autonomous region | 106.24 | 36.02 | Wang, 2009; Wang, 2011; Su, 2013 |
| China | Guyuan, Ningxia autonomous region | 106.64 | 35.85 |  |
| China | Hanzhong, Shaanxi province | 107.04 | 33.08 | Wang et al., 2008 |
| China | Hengshui, Hebei province | 115.67 | 37.74 | Zhang et al., 2014 |
| China | Huaibei region, Anhui province | 116.79 | 33.95 | Gao et al., 2015 |
| China | Huanglong county, Shaanxi province | 109.83 | 35.58 | Xiao et al., 2008 |
| China | Jining, Shandong province | 116.58 | 35.42 | Zhao et al., 2005 |
| China | Kuerle, Xinjiang autonomous region | 84.25 | 41.78 | Yu et al., 2012 |
| China | Lanzhou, Gansu province | 103.83 | 36.06 | Zhang et al., 2001; Chen, 2013 |
| China | Liaocheng, Shandong province | 116.02 | 36.46 | Gao et al., 2015; Ren et al., 2007 |
| China | Liaocheng, Shandong province | 115.96 | 36.44 |  |
| China | Liaocheng, Shandong province | 115.85 | 36.75 |  |
| China | Liaocheng, Shandong province | 115.99 | 36.43 |  |
| China | Lingwu, Ningxia autonomous region | 106.34 | 38.1 | Yuan et al., 2010; Su, 2013; Yu, 2013 |
| China | Lingwu, Ningxia autonomous region | 106.34 | 38.06 |  |
| China | Lingwu, Ningxia autonomous region | 106.34 | 38.11 |  |
| China | Lingwu, Ningxia autonomous region | 106.3 | 38.12 |  |
| China | Lingwu, Ningxia autonomous region | 106.34 | 38.22 |  |
| China | Minhe, Qinghai province | 102.92 | 36.19 | Zhang, 2007 |
| China | Minhe, Qinghai province | 102.86 | 36.14 |  |
| China | Minhe, Qinghai province | 102.8 | 36.33 |  |
| China | Minhe, Qinghai province | 102.84 | 35.88 |  |
| China | Minhe, Qinghai province | 102.81 | 35.88 |  |
| China | Qinling, Shaanxi province | 108.76 | 36.82 | Xiao et al., 2008 |
| China | Shanghai | 121.47 | 31.23 | Zhang et al., 2001; Chen, 2013 |
| China | Shenyang, Liaoning province | 123.31 | 41.8 | Tong, 2001 |
| China | Shijiazhuang, Hebei province | 115.57 | 37.74 | Kang et al., 2010; Liu et al., 2010 |
| China | Shijiazhuang, Hebei province | 114.51 | 38.04 |  |
| China | Shijiazhuang, Hebei province | 114.38 | 38.11 |  |
| China | Shijiazhuang, Hebei province | 114.46 | 38.14 |  |
| China | Shizuishan, Ningxia autonomous region | 106.52 | 38.91 | Song et al., 2012; Zhang, 2001 |
| China | Shizuishan, Ningxia autonomous region | 106.38 | 38.98 |  |
| China | Shizuishan, Ningxia autonomous region | 106.36 | 39.04 |  |
| China | Sichuan province | 104.06 | 30.57 | Zhang, 2007 |
| China | Tangshan, Hebei province | 117.81 | 39.88 | An et al., 2010 |
| China | Tianjin | 117.4 | 40.04 | Zhang et al., 2014; Hu et al., 2005 |
| China | Tianjin | 117.04 | 39.38 |  |
| China | Tianjin | 116.97 | 38.95 |  |
| China | Tianjin | 117.28 | 39.07 |  |
| China | Tianjin | 117.13 | 39.22 |  |
| China | Tianjin | 117.38 | 39.75 |  |
| China | Tianjin | 117.12 | 39.05 |  |
| China | Tianjin | 117.8 | 39.24 |  |
| China | Tianjin | 117.47 | 38.86 |  |
| China | Tianjin | 117.31 | 39.09 |  |
| China | Wuhan, Hubei province | 114.3 | 30.6 | Yu et al., 2012 |
| China | Wuqi county, Shaanxi province | 108.17 | 36.92 | Xiao et al., 2008 |
| China | Wuzhong, Ningxia autonomous region | 106.19 | 38 | Yu, 2013 |
| China | Xingtai, Hebei province | 114.5 | 37.07 | Zhang et al., 2014 |
| China | Xuchang, Henan province | 114.17 | 34.1 | Chen et al., 2009 |
| China | Xuchang, Henan province | 113.82 | 34.05 |  |
| China | Xuchang, Henan province | 113.76 | 34.22 |  |
| China | Xunhua, Qinghai province | 102.46 | 35.71 | Jia et al., 2012 |
| China | Xuzhou, Jiangsu province | 117.19 | 34.18 | Zhang et al., 2001; Chen, 2013 |
| China | Yantai, Shandong province | 119.96 | 37.13 | Zhao et al., 2005 |
| China | Yinchuan, Ningxia autonomous region | 106.33 | 38.67 | Zhang, 2001; Yuan et al., 2010; Su, 2013; Yu, 2013 |
| China | Yinchuan, Ningxia autonomous region | 106.15 | 38.5 |  |
| China | Yinchuan, Ningxia autonomous region | 106.31 | 38.45 |  |
| China | Yinchuan, Ningxia autonomous region | 106.14 | 38.68 |  |
| China | Yinchuan, Ningxia autonomous region | 106.28 | 38.46 |  |
| China | Yongning, Ningxia autonomous region | 106.2 | 38.22 | Sun, 2011; Li, 2010 |
| China | Yongshou county, Shaanxi province | 108.14 | 34.69 | Wang et al., 2008 |
| China | Yu County, Hebei province | 114.58 | 39.84 | Yang et al., 2013 |
| China | Yuncheng, Shanxi province | 111.03 | 35.05 | Fan et al., 2014 |
| China | Yuzhou, Henan province | 113.48 | 34.14 | Chen et al., 2009 |
| China | Zhengzhou, Henan province | 113.66 | 34.76 | Qin, 1996 |
| China | Zhidan county, Shaanxi province | 109.35 | 36.28 | Xiao et al., 2008 |
| China | Zhongwei, Ningxia autonomous region | 105.19 | 37.5 | Sun, 2011; Li, 2010 |
| China | Zhongwei, Ningxia autonomous region | 105.73 | 37.46 |  |
| China | Zhongwei, Ningxia autonomous region | 105.77 | 37.5 |  |
| China | Zhongwei, Ningxia autonomous region | 105.75 | 37.49 |  |
| China | Inter mongolia autonomous region | no detailed | | Liu et al., 2013 |
| China | Fujian province | no detailed | | Zhang et al., 2001; Zhao & Chen, 1980; Chen, 2013 |
| China | Zhejiang province | no detailed | | Zhang et al., 2001; Zhao & Chen, 1980; Chen, 2013 |

**Table S3: Summary of the detailed distribution sites of EBR used to the potential distributions prediction.**

| **Country** | **Locations** | **Longitude** | **Latitude** | **References** |
| --- | --- | --- | --- | --- |
| China | Akesu, Xinjiang autonomous region | 80.22 | 41.13 | Zhou, 2013 |
| China | Akesu, Xinjiang autonomous region | 80.27 | 41.17 |
| China | Akesu, Xinjiang autonomous region | 80.27 | 41.16 |
| China | Akesu, Xinjiang autonomous region | 80.31 | 41.15 |
| China | Akesu, Xinjiang autonomous region | 81.15 | 40.34 |
| China | Anqing, Anhui province | 117.11 | 30.79 | Ge, 2014; Zhang, 2007; Yuan et al., 2013; Ren, 2000 |
| China | Anyang, Henan province | 114.39 | 36.10 | Qin, 1996 |
| China | Baoji, Shaanxi province | 107.24 | 34.36 | Yu, 2012; Wang et al., 2012; Li&Tang, 1980 |
| China | Beijing | 116.10 | 39.94 | Yu, 2012; Tong et al., 1993; Huang et al., 2012; Yu, 2011; Hu et al.,2012 |
| China | Beijing | 116.21 | 39.99 |
| China | Beijing | 116.22 | 39.91 |
| China | Beijing | 116.26 | 40.25 |
| China | Beijing | 116.32 | 39.94 |
| China | Beijing | 116.34 | 40.01 |
| China | Beijing | 116.39 | 40.02 |
| China | Beijing | 116.48 | 39.80 |
| China | Beijing | 116.84 | 40.37 |
| China | Binzhou, Shandong province | 117.48 | 37.22 | Shang, 2013 |
| China | Changsha, Hunan province | 112.94 | 28.23 | Zhang et al., 2009 |
| China | Dalian, Liaoning province | 121.86 | 39.06 | Xu, 2008 |
| China | Datong, Shanxi province | 113.17 | 40.04 | Yuan, 2015 |
| China | Datong, Shanxi province | 113.17 | 40.02 |
| China | Datong, Shanxi province | 113.18 | 40.04 |
| China | Datong, Shanxi province | 113.22 | 40.00 |
| China | Fuxian, Shaanxi province | 109.38 | 35.99 | Xiao, 2008 |
| China | Fuyang, Anhui province | 115.64 | 32.57 |
| China | Fuyang, Anhui province | 115.81 | 32.89 |
| China | Fuyang, Anhui province | 116.58 | 30.69 | Yu, 2012 |
| China | Ganquan, Shaanxi province | 109.35 | 36.28 | Xiao, 2008 |
| China | Guyuan, Ningxia autonomous region | 106.12 | 35.62 | Wang, 2009; Wang, 2011; Su, 2013 |
| China | Guyuan, Ningxia autonomous region | 106.28 | 36.02 |
| China | Guyuan, Ningxia autonomous region | 106.29 | 36.00 |
| China | Guyuan, Ningxia autonomous region | 106.64 | 35.85 |
| China | Hanzhong, Shaanxi province | 107.04 | 33.08 | Yu, 2012; Wang et al., 2012; Li&Tang, 1980 |
| China | Harbin, Heilongjiang province | 126.66 | 45.74 | Ren et al., 2000 |
| China | Hengshui, Hebei province | 115.65 | 37.65 | Bai, 2012; Kang et al., 2010; Liu et al., 2010; Qiao et al., 2011 |
| China | Hengshui, Hebei province | 115.67 | 37.74 |  |
| China | Huaibei region, Anhui province | 116.79 | 33.95 | Ge, 2000 |
| China | Huanglong, Shaanxi province | 109.84 | 35.58 | Xiao, 2008 |
| China | Huludao, Liaoning province | 119.94 | 40.73 | Yu, 2012 |
| China | Jilin province | 126.55 | 43.84 | Ren et al., 2000 |
| China | Jincheng, Shanxi province | 112.73 | 37.69 | Wang et al., 2013 |
| China | Jining, Shandong province | 116.34 | 35.41 | Zhao, 2005; Yu, 2013 |
| China | Jining, Shandong province | 116.62 | 35.40 |
| China | Lanzhou, Gansu province | 103.63 | 36.09 | Zhang, 2001; Hu et al., 2011 |
| China | Lanzhou, Gansu province | 103.83 | 36.06 |
| China | Lianyungang, Jiangsu province | 119.30 | 34.60 | Jiang, 1990 |
| China | Liaocheng, Shandong province | 115.85 | 36.75 | Ren, 2004 |
| China | Liaocheng, Shandong province | 115.96 | 36.44 |  |
| China | Liaocheng, Shandong province | 116.03 | 36.46 |  |
| China | Lingwu, Ningxia autonomous region | 106.34 | 38.10 | Wang, 2009; Wang, 2011; Su, 2013 |
| China | Luochuan, Shaanxi province | 109.43 | 35.76 | Xiao, 2008 |
| China | Mengcheng, Anhui province | 116.37 | 33.39 | Ge, 2000 |
| China | Mengcheng, Anhui province | 116.38 | 33.39 |
| China | Minhe, Qinghai province | 102.80 | 36.33 | Zhang, 2007 |
| China | Minhe, Qinghai province | 102.81 | 35.88 |
| China | Minhe, Qinghai province | 102.84 | 35.88 |
| China | Minhe, Qinghai province | 102.86 | 36.14 |
| China | Minhe, Qinghai province | 102.92 | 36.19 |
| China | Neixiang, Henan province | 111.84 | 33.05 | Chen, 2009; Cao et al., 2012 |
| China | Qingdao, Shandong province | 120.03 | 37.16 | Ding et al., 2010 |
| China | Qingdao, Shandong province | 120.38 | 36.07 |
| China | Shanghai | 121.47 | 31.23 | Zhang, 2007; Yuan, 2013 |
| China | Shenyang, Liaoning province | 123.32 | 41.80 | Tong, 2001; Hu, 2011 |
| China | Shenyang, Liaoning province | 123.43 | 41.84 |
| China | Shijiazhuang, Hebei province | 114.34 | 38.15 | Bai, 2012; Kang et al., 2010; Liu et al., 2010; Qiao et al., 2011 |
| China | Shijiazhuang, Hebei province | 114.42 | 38.02 |
| China | Shijiazhuang, Hebei province | 114.45 | 38.05 |
| China | Shijiazhuang, Hebei province | 114.46 | 38.08 |
| China | Shijiazhuang, Hebei province | 114.46 | 38.14 |
| China | Shijiazhuang, Hebei province | 114.49 | 38.09 |
| China | Shijiazhuang, Hebei province | 114.49 | 38.08 |
| China | Shijiazhuang, Hebei province | 114.51 | 38.04 |
| China | Shijiazhuang, Hebei province | 114.52 | 38.04 |
| China | Shijiazhuang, Hebei province | 114.54 | 38.02 |
| China | Shizuishan, Ningxia autonomous region | 106.36 | 39.04 | Song et al., 2012; Zhang, 2001 |
| China | Shizuishan, Ningxia autonomous region | 106.38 | 38.98 |
| China | Shizuishan, Ningxia autonomous region | 106.52 | 38.91 |
| China | Shizuishan, Ningxia autonomous region | 106.57 | 38.63 |
| China | Shunping, Hebei province | 115.07 | 38.92 | Bai, 2012; Kang et al., 2010; Liu et al., 2010; Qiao et al., 2011 |
| China | Sichuan province | 104.06 | 30.57 | Zhang, 2007 |
| China | Sichuan province | 104.08 | 30.65 |
| China | Taian, Shandong province | 116.47 | 35.94 | Sun, 1992; Yu, 2013 |
| China | Taian, Shandong province | 116.73 | 36.27 |
| China | Taian, Shandong province | 117.16 | 36.05 |
| China | Taian, Shandong province | 117.18 | 35.91 |
| China | Tangshan, Hebei province | 117.82 | 39.88 | Wu&Zhao, 2006; An et al., 2010; Chu et al., 2013 |
| China | Tianjin | 116.97 | 38.95 | Hu et al., 2005; Li et al., 2007; Liu et al., 2013; Lei et al., 2013 |
| China | Tianjin | 117.04 | 39.38 |
| China | Tianjin | 117.12 | 39.06 |
| China | Tianjin | 117.14 | 39.22 |
| China | Tianjin | 117.21 | 39.17 |
| China | Tianjin | 117.31 | 39.09 |
| China | Tianjin | 117.38 | 39.75 |
| China | Tianjin | 117.44 | 40.01 |
| China | Tianjin | 117.47 | 38.84 |
| China | Tianjin | 117.52 | 38.91 |
| China | Tianjin | 117.71 | 39.00 |
| China | Tianjin | 117.77 | 39.21 |
| China | Tianjin | 117.82 | 39.33 |
| China | Tieling, Liaoning province | 123.84 | 42.29 | Tong, 2001; Hu, 2011 |
| China | Weifang, Shandong province | 119.16 | 36.71 | Ding et al., 2010 |
| China | Weihai, Shandong province | 122.12 | 37.51 | Ding et al., 2010 |
| China | Wuhan, Hubei province | 114.31 | 30.59 | Zhang, 2007; Yu, 2012 |
| China | Wuqi, Shaanxi province | 108.18 | 36.92 | Wang et al., 2008; Wang&Wang, 2010 |
| China | Wuzhong, Ningxia autonomous region | 106.20 | 38.00 | Ma, 2009; Wu, 2011 |
| China | Xian, Shaanxi province | 108.95 | 34.27 | Yu, 2012; Wang et al., 2012; Li&Tang, 1980 |
| China | Xian, Shaanxi province | 108.96 | 34.27 |
| China | Xian, Shaanxi province | 108.99 | 34.25 |
| China | Xian, Shaanxi province | 109.24 | 34.38 |
| China | Xianyang, Shaanxi province | 108.14 | 34.69 | Wang et al., 2008; Wang&Wang, 2010 |
| China | Xingping, Shaanxi province | 108.49 | 34.30 | Wang et al., 2008; Wang&Wang, 2010 |
| China | Xingtai, Hebei province | 114.50 | 37.07 | Bai, 2012; Kang et al., 2010; Liu et al., 2010; Qiao et al., 2011 |
| China | Xuchang, Henan province | 113.49 | 34.14 | Chen, 2009; Cao et al., 2012 |
| China | Xuchang, Henan province | 113.77 | 34.22 |
| China | Xuchang, Henan province | 113.83 | 34.05 |
| China | Xuchang, Henan province | 114.18 | 34.10 |
| China | Xunhua, Qinghai province | 102.46 | 35.71 | Jia et al., 2012 |
| China | Xuzhou, Jiangsu province | 117.16 | 34.21 | Yu, 2012; Ding&Hu, 2009 |
| China | Xuzhou, Jiangsu province | 117.19 | 34.18 |  |
| China | Yanan, Shaanxi province | 108.77 | 36.82 | Xiao, 2008 |
| China | Yantai, Shandong province | 120.78 | 37.44 | Qin et al., 1999; Niu, 2011 |
| China | Yinchuan, Ningxia autonomous region | 106.04 | 38.61 | Zhang, 2001; Yuan et al., 2010; Su, 2013; Yu, 2013 |
| China | Yinchuan, Ningxia autonomous region | 106.13 | 38.50 |
| China | Yinchuan, Ningxia autonomous region | 106.16 | 38.50 |
| China | Yinchuan, Ningxia autonomous region | 106.24 | 38.47 |
| China | Yinchuan, Ningxia autonomous region | 106.26 | 38.47 |
| China | Yinchuan, Ningxia autonomous region | 106.28 | 38.43 |
| China | Yinchuan, Ningxia autonomous region | 106.29 | 38.47 |
| China | Yinchuan, Ningxia autonomous region | 106.33 | 38.45 |
| China | Yinchuan, Ningxia autonomous region | 106.35 | 38.55 |
| China | Yongning, Ningxia autonomous region | 106.25 | 38.28 | Sun, 2011; Li, 2010 |
| China | Yuncheng, Shanxi province | 110.77 | 35.14 | Fan et al., 2014 |
| China | Yuncheng, Shanxi province | 111.03 | 35.05 |
| China | Yuxian, Hebei province | 114.59 | 39.84 | Zhao&Cui, 2008; Huang et al., 2014 |
| China | Zhangjiakou, Hebei province | 114.89 | 40.82 | Zhao&Cui, 2008; Huang et al., 2014 |
| China | Zhengzhou, Henan province | 113.66 | 34.76 | Chen, 2009; Cao et al., 2012 |
| China | Zhongwei, Ningxia autonomous region | 105.67 | 37.55 | Sun, 2011; Li, 2010 |
| China | Zhongwei, Ningxia autonomous region | 105.69 | 37.48 |
| China | Zhongwei, Ningxia autonomous region | 105.73 | 37.47 |
| China | Zhongwei, Ningxia autonomous region | 105.75 | 37.49 |
| China | Zhongwei, Ningxia autonomous region | 105.77 | 37.50 |
| China | Inter mongolia autonomous region | no detailed | | Liu, 2013 |
| Japan |  | no detailed | | Borovec，2013 |
| North Korea |  | no detailed | | Borovec，2013 |
| Far East |  | no detailed | | Zhao & Chen, 1980 |
